# Linking *In Vitro* and *In Vivo* Survival of Clinical *Leishmania donovani* Strains

**DOI:** 10.1371/journal.pone.0012211

**Published:** 2010-08-17

**Authors:** Manu Vanaerschot, Ilse Maes, Meriem Ouakad, Vanessa Adaui, Louis Maes, Simonne De Doncker, Suman Rijal, François Chappuis, Jean-Claude Dujardin, Saskia Decuypere

**Affiliations:** 1 Unit of Molecular Parasitology, Department of Parasitology, Institute of Tropical Medicine, Antwerp, Belgium; 2 Laboratory for Microbiology, Parasitology and Hygiene, Department of Biomedical Sciences, Antwerp University, Antwerp, Belgium; 3 Institute of Tropical Medicine ‘Alexander von Humboldt’, Universidad Peruana Cayetano Heredia, Lima, Peru; 4 Department of Internal Medicine, B.P. Koirala Institute of Health Sciences, Dharan, Nepal; 5 Division of International and Humanitarian Medicine, Geneva University Hospitals and University of Geneva, Geneva, Switzerland; Federal University of São Paulo, Brazil

## Abstract

**Background:**

*Leishmania donovani* is an intracellular protozoan parasite that causes a lethal systemic disease, visceral leishmaniasis (VL), and is transmitted between mammalian hosts by phlebotomine sandflies. *Leishmania* expertly survives in these ‘hostile’ environments with a unique redox system protecting against oxidative damage, and host manipulation skills suppressing oxidative outbursts of the mammalian host. Treating patients imposes an additional stress on the parasite and sodium stibogluconate (SSG) was used for over 70 years in the Indian subcontinent.

**Methodology/Principal Findings:**

We evaluated whether the survival capacity of clinical *L. donovani* isolates varies significantly at different stages of their life cycle by comparing proliferation, oxidative stress tolerance and infection capacity of 3 Nepalese *L*. *donovani* strains in several *in vitro* and *in vivo* models. In general, the two strains that were resistant to SSG, a stress encountered in patients, attained stationary phase at a higher parasite density, contained a higher amount of metacyclic parasites and had a greater capacity to cause *in vivo* infection in mice compared to the SSG-sensitive strain.

**Conclusions/Significance:**

The 2 SSG-resistant strains had superior survival skills as promastigotes and as amastigotes compared to the SSG-sensitive strain. These results could indicate that *Leishmania* parasites adapting successfully to antimonial drug pressure acquire an overall increased fitness, which stands in contrast to what is found for other organisms, where drug resistance is usually linked to a fitness cost. Further validation experiments are under way to verify this hypothesis.

## Introduction

Visceral leishmaniasis (VL), also known as Kala-Azar in the Indian subcontinent, is a lethal systemic disease caused by intracellular protozoan parasites of the *Leishmania donovani* complex. India, Nepal and Bangladesh carry 67.3% of the global VL disease burden [1], [2]. *Leishmania* is transmitted between mammalian hosts by phlebotomine sandflies and accordingly grows in 2 life-forms (i) extracellular promastigotes which are adapted to the sandfly and (ii) intracellular amastigotes adapted to macrophages of the host [3]. The first step of the differentiation from promastigotes into amastigotes is the transformation from non-infective dividing promastigotes into infective ‘metacyclic’ promastigotes in the sandfly through a process called metacyclogenesis [4]. Once transmitted to the host, metacyclic promastigotes are phagocytised by macrophages and transform into amastigotes.

Throughout its natural life cycle, *Leishmania* encounters hostile conditions including (i) oxidative stress due to heme digestion in the blood meal [5] and midgut proteases in the sandfly [6], (ii) complement lysis in the blood upon transmission [7] and (iii) reactive oxygen and nitrogen species (ROS and RNS) when phagocytised by macrophages of the host [8]. *Leishmania* successfully adapted to these different environments for thousands of years and developed a highly adaptive character. Their survival capacity relies mainly on a unique oxidant protective redox metabolism [9], and skills to suppress oxidative outbursts of the host defence mechanisms [10]–[13]. In the last hundred years, *Leishmania* parasites have encountered an additional stress imposed by the treatment of patients. However, the decreasing efficacy of antimonial treatment (SSG) and the linked emergence of antimonial parasite resistance in the Indian subcontinent proofs that the parasite also managed to adapt to this drug pressure [14], [15].

In this work, we evaluate whether the survival capacity of clinical *L. donovani* isolates varies significantly at different stages of their life cycle. Susceptibility to various stresses, *in vitro* growth profile and *in vivo* infection burden in mice were comparatively studied in 3 Nepalese strains. The results of this model are discussed in relation to the strains' SSG-tolerance and the potential impact on their general fitness.

## Materials and Methods

### Patients and parasites

The *L. (L.) donovani* strains MHOM/NP/03/BPK282/0, MHOM/NP/02/BPK085/0 and MPHOM/NP/03/BPK275/0 were isolated from bone marrow aspirates from confirmed VL patients recruited at the B.P. Koirala Institute of Health Sciences, Dharan, Nepal [16]. Written informed consent was obtained from the patients and in the case of children, from the parents or guardians. Ethical clearance was obtained from the institutional review boards of the Nepal Health Research Council, Kathmandu, Nepal, and the Institute of Tropical Medicine, Antwerp, Belgium.

The patients received a full supervised treatment course of sodium antimony gluconate (SAG) (Albert David Ltd, Calcutta) of 20 mg/kg/day i.m. for 30 days and were followed-up for clinical and parasitological evaluation at the end of treatment and at 3, 6 and 12 months after start of treatment. Definite cure (BPK282/0 and BPK085/0) was defined as a patient with initial cure who showed no symptoms and signs of relapse at the 12 months follow-up visit. Non-responders (BPK275/0) were cases with positive parasitology of a bone marrow aspirate after a full 30 days SAG drug course. The clinical isolates were identified as *L. donovani* by PCR-RFLP analysis of cysteine proteinase b [17] and were further characterised as members of the same genomic subpopulation which is circulating in most leishmaniasis endemic regions in Nepal [18]. To obtain homogenous working parasite populations, the 3 isolates were cloned using the micro-drop method [19] resulting in respectively BPK282/0cl4, BPK085/0cl3 and BPK275/0cl12.

### *In vitro* promastigote cultures

For routine cultures, promastigotes were grown on modified Eagle's medium or HOMEM [20] (Invitrogen) supplemented with 20% (v/v) heat-inactivated foetal calf serum (FCS) (PAA Laboratories GmbH), pH 7.5, at 26°C for 7 days. The cultures were initiated by inoculating 3^rd^ to 4^th^ day stationary phase parasites in 5 ml of culture medium at a final concentration of 5×10^5^ parasites per ml. Before or during each experiment, parasite density was microscopically determined every 24 hours using Uriglass disposable count chambers (Menarini diagnostics) to follow up the growth and differentiation profile.

### *In vitro* promastigote growth profile and starvation tests

Third to 4^th^ day stationary phase promastigotes from routine cultures were inoculated in 51 ml at a final concentration of 5×10^5^ parasites per ml and grown in the following conditions: (i) HOMEM supplemented with 20% FCS incubated at 26°C as control condition, (ii) modified Eagle's medium supplemented with 0%, 1%, 5% or 10% FCS incubated at 26°C and (iii) PBS incubated at 26°C. Parasite density was microscopically determined every 24 hours for 7 to 14 days. All experiments with the different growth conditions were repeated 3 times and GraphPad Prism 5 was used to perform statistical analysis using the two-way ANOVA test, the Akaike's information criterion and to visualise results.

### *In vitro* promastigote stress tests

Promastigotes were exposed to different stresses (Table 1) at late log phase, 1^st^ day stationary phase and 2^nd^ day stationary phase according to the concomitantly determined growth curve to evaluate whether tolerance to stress changes throughout promastigote growth. Parasites were counted and diluted to 4×10^6^ or 5×10^5^ parasites per ml for 24 hours and 48 hours experiments respectively, and 100 µl was plated out in quadruplicate on sterile 96 well plates (Corning Costar). Each well was topped with 100 µl drug solution, which was prepared in 6 different concentrations. One untreated control and 1 blank per drug per strain were plated out in quadruplicate along the treated wells. The plates were sealed and incubated at 26°C for 24 or 48 hours. Resazurin (Invitrogen) was added after 1 hour in case of 24 hours drug-exposure, or after 24 hours in case of 48 hours exposure. Another 24 hours later, growth inhibition was evaluated by measuring fluorescence using the Victor X3 Multilabel Reader (PerkinElmer) excitating at 560 nm and measuring emission at 590 nm. All experiments with the different conditions were repeated 3 times. The blank-subtracted data expressed in relative fluorescence units was exported to GraphPad Prism 5 to calculate the 50% inhibitory concentration (IC_50_) using a sigmodial dose-response model with variable slope, to visualise results and to statistically analyse the data with a two-way ANOVA test with Bonferroni post tests.

**Table 1:** List of drugs used for promastigote and amastigote stress tests.

Compound	Life stage tested	Incubation time	Concentration range
S-nitroso-N-acetyl-DL-penicillamine^1^ (SNAP)	promastigotes	48 hours	5.00 µM–500.00 µM
	amastigotes	24 hours	20.00 µM–800.00 µM
3-morpholinosydnonimine^2^ (SIN1)	promastigotes	24 hours	0.03 mM–9.00 mM
hydrogen peroxide^1^ (H_2_O_2_)	promastigotes	24 hours	305.55 µM–9777.46 µM
copper sulphate pentahydrate^1^	promastigotes	24 hours	3.47 µg/ml–1124.68 µg/ml
potassium dichromate^1^	promastigotes	24 hours	31.62 pg/ml–3.16 µg/ml
potassium antimonyl tartrate trihydrate^1^ (SbIII)	promastigotes	24 hours	20.00 µg/ml–7.00 mg/ml
Sodium stibogluconate^3^ (SSG)	amastigotes	24 hours	3.00 – 60.00 µg/ml

1 purchased at Sigma-Aldrich,

2 purchased at Biomol,

3 obtained additive free from GlaxoSmithKline.

### *In vitro* amastigote infection and stress tests

For all infection and amastigote stress tests, primary peritoneal macrophages were extracted from Swiss OF1-mice (Charles River) and plated out at 2×10^5^ cells per 200 µl per well in RPMI supplemented with 10% FCS and 100 U/ml penicillin and streptomycin ( =  full RPMI) in 16 well Lab-Tek chamber slides (Nunc) incubated at 37°C+5% CO_2_. Twenty-four hours later, medium was gently removed and the macrophages attached to the slide were infected with 2^nd^ or 3^rd^ day stationary phase promastigotes at a ratio of 7 parasites per macrophage in a volume of 200 µl full RPMI. After 24 hours of infection, non-internalised parasites were washed off and SNAP dilutions (7 concentrations + negative controls) with or without SSG or SSG dilutions (4 concentrations + negative controls) were applied in quadruplicate. For all tests with SNAP, drug solutions were removed after 24 hours and full RPMI was added for another 48 hours. For tests with SSG only, drug solutions were refreshed after 48 hours, and exposure lasted 72 hours. Finally, slides were fixated with methanol and stained with 1/10 GIEMSA. For all tests, infection level was evaluated by counting the percentage of infected macrophages and the number of parasites per infected macrophage and these 2 factors were multiplied to obtain the infection index. All details on the drugs and concentrations used in these experiments are summarised in Table 1.

The data was exported to GraphPad Prism 5 to calculate ED_50_'s using a sigmodial dose-response model with variable slope, to comparatively analyse the data with one way ANOVA and to visualise results.

### *In vivo* infection and sampling

Female Balb/c mice were purchased from Charles River and infected at 6 to 8 weeks of age. Ninety-eight mice were used to monitor the *in vivo* infection of the 3 chosen parasite strains at 7 time points: four mice per time point per strain were intraperitoneally injected with 10^7^ parasites of 2^nd^ to 3^rd^ day stationary phase cultures diluted in 100 µl PBS and 2 control mice per time point were intraperitoneally injected with 100 µl PBS. The intraperitoneal route of infection was preferred above the intravenous route to improve the homogeneity in results as shown by others with *L. infantum* [21]. Mice were sacrificed at day 3, 7, 14, 28, 42, 56 and 70 post infection to sample liver, spleen and bone marrow. Livers and spleens were homogenised in a volume of respectively 1.5 ml and 0.75 ml RPMI supplemented with 100 units penicillin/streptomycin per ml. Bone marrow was extracted by repeatedly flushing the left femur with 1 ml of the same solution. Eighty µl of homogenised spleens and livers and 400 µl of extracted bone marrow were sampled and used for subsequent tissue lysis and DNA extraction.

Mouse care and experimental procedures were performed under approval of the Animal Ethic Committee of the Institute of Tropical Medicine Antwerp (PAR-018/2).

### Tissue lysis and DNA extraction

Liver and spleen samples were lysed and DNA was extracted using the QIAamp DNA mini kit according to the manufacturer's instructions (tissue protocol). Bone marrow samples were processed with a similar protocol, but using double volumes of buffer AL and ethanol. The concentration of the isolated DNA was assessed by spectrophotometry using the Nanodrop ND-1000 (Isogen life science).

### Real-time quantitative PCR and analysis

Quantification of the parasite load in murine tissues was done with 2 separate quantitative PCRs (QPCRs): (i) one PCR targeting kDNA to quantify *Leishmania* as described by Nicolas et al [22] and (ii) one PCR targeting the single copy gene neurotrophin 3 of mice to quantify the amount of murine cells present in each sample (Table 2). The conditions for the *Leishmania* PCR were optimised and a standard curve of serial diluted samples, consisting of murine tissue mixed with known quantities of *L. donovani*, showed a linear signal between 1 and 4×10^5^ parasites in a 80 µl sample. The mouse primers gave a linear signal between 1,3 and 106 ng/µl mouse DNA. QPCRs were run with the iQ Sybr Green Supermix (Bio-Rad), 2 µL of DNA, and the primers in a 25 µl format with the following thermal profile: (i) initial denaturation for 5 min at 95°C, (ii) 36 cycles of denaturation for 30 s at 95°C, annealing for 15 s at 58,5°C or 65°C (depending on target) and extension for 15 s at 72°C. Melt curve analysis was done at the end of all QPCRs to verify PCR product quality and primer dimer formation.

**Table 2:** Primers used for quantification of *L. donovani* and mouse cells.

	Target	Primers	Sequence	Concentration in QPCR	Annealing temperature
*Leishmania*	kDNA	JW11^1^	5′-CCTATTTTACACCAACCCCCAGT-3′	250 nM	58.5°C
		JW12^1^	5′-GGGTAGGGGCGTTCTGCGAAA-3′	250 nM	
Mouse	ntf3^2^	NTF3-F	5′-ACTTCGCAAACCTATGTCCG-3′	400 nM	65.0°C
		NTF3-R	5′-CCAATTTTTCTCGACAAGGC-3′	400 nM	

1 described by Nicolas et al, 2000;

2 NM_008742

All QPCRs were run on the iCycler (Bio-Rad) in 96 well plates; technical variation between plates was minimised by including 2 calibrators on each plate. If a given sample had a total DNA concentration higher then 106 ng/µl or if the Ct-value exceeded the linear range, the sample was diluted and both the *Leishmania* and the mouse QPCR were repeated.

Raw data was imported into qbase^plus^ (Biogazelle) [23] for calibration between plates and normalisation of the *Leishmania* signal to the mouse signal. The infection level is thus described as a normalised amount of *Leishmania* per fixed unit of mouse cells, which allows comparison of parasite load between tissues.

The resulting calibrated normalised relative quantities were exported to GraphPad Prism 5 for statistical analysis using two-way ANOVA with Bonferroni post tests and visualisation of the results.

## Results and Discussion

### *In vitro* promastigote tests

#### Growth profile

**Figure 1:**
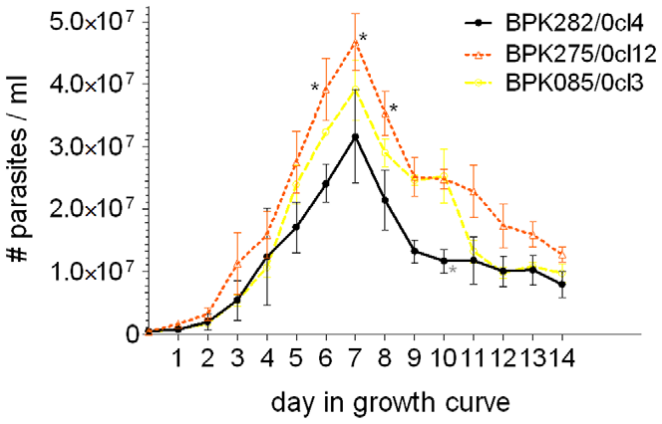
Growth profile of cultured promastigotes in routine conditions for 14 days. Error bars indicate standard error of the mean based on 3 repeated experiments, black stars indicate a significant difference with BPK282/0cl4 and grey stars with BPK085/0cl3 at a specific time point. This graph shows that BPK275/0 reaches stationary phase at a higher parasite density compared to BPK282/0cl4. There were no significant differences between BPK085/0cl3 and BPK275/0cl12.

The parasite density in routine culture conditions was evaluated every day for 14 days to study and compare the growth features of the three strains as promastigotes. In general, we found that BPK282/0cl4 reaches the stationary phase at a lower parasite density compared to the two other strains, BPK085/0cl3 being an intermediate (Fig. 1). Major factors determining entry into stationary phase may be (i) pH of the medium, (ii) availability of nutrients and (iii) quorum sensing. Parasite multiplication in culture causes consumption of nutrients resulting in nutrient scarceness and medium acidification, conditions that are known to induce metacyclogenesis [24]. Quorum sensing allows a population of individuals to coordinate global behaviour and as such act as a multi-cellular unit [25]. A convergence in pathways of quorum and starvation sensing is suggested to regulate entry into stationary phase of bacterial growth [26]. A density sensing mechanism has already been identified in *Trypanosoma brucei* [27]; possibly a similar mechanism exists in *Leishmania*. The differential growth profiles observed here could thus be due to differences in (i) metabolic requirements, (ii) tolerance to starvation and acidification, or (ii) some form of quorum sensing. Regardless the underlying cause, our results indicate that BPK275/0cl12 might have an advantage in terms of transmission to the host. If the observed differential *in vitro* growth reflects parasite proliferation in the sandfly, then more parasites would be transmitted to the host through the same inoculum volume of BPK275/0cl12 compared to BPK282/0cl4. However, this advantage will only hold if the percentage of infective metacyclic parasites is similar or higher in the inoculum of BPK275/0cl12 compared to BPK282/0cl4. This will be evaluated in the *in vitro* infectivity study.

#### Starvation and temperature stresses

**Figure 2:**
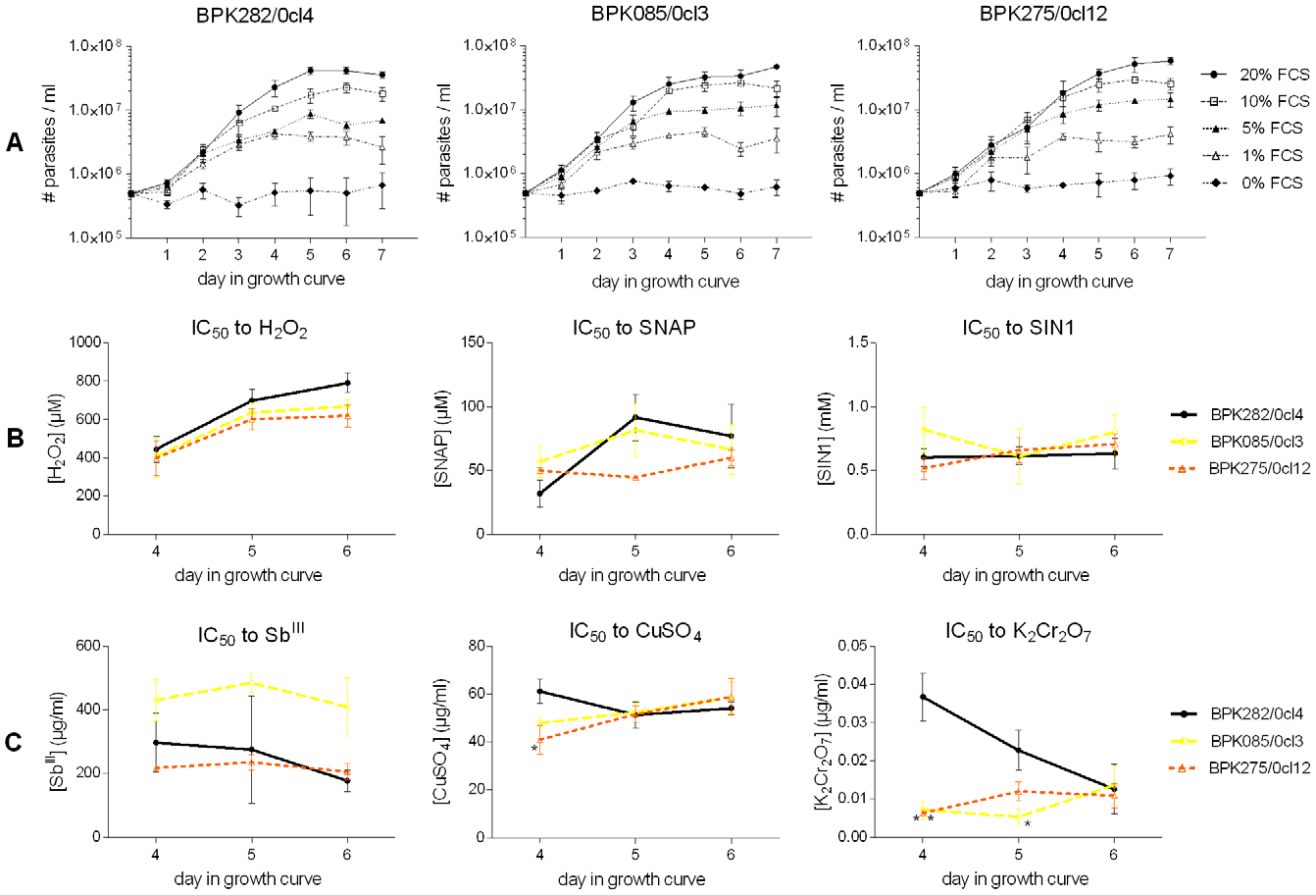
Promastigote growth curves in stress conditions. Row A: starvation conditions, row B: oxidative stress tests and row C: metal stress tests. The outcome of the stress tests is expressed in IC_50_ over time. Stars indicate statistically significant differences in the stress tests compared to BPK282/0cl4. Error bars indicate the standard error of the mean based on 3 repeated experiments. The data on oxidative and metal stress tolerance is available as supplementary material (Table S1). All strains had an equal tolerance to starvation conditions, H_2_O_2_, SNAP, SIN1 and Sb^III^. All strains showed a significant increase in tolerance to H_2_O_2_ towards the stationary phase (p = 0.002). Significant differences were found in tolerance to CuSO_4_ at day 4 between BPK282/0cl4 and BPK275/0cl12, in tolerance to K_2_Cr_2_O_7_ between BPK282/0cl4 and the two other strains at day 4 and between BPK282/0cl4 and BPK085/0cl3 at day 5.

In the previous experiment, BPK275/0cl12 reached higher parasite densities having the same amount of nutrients available as the other strains. We further evaluated the proliferation capacity of the strains in starvation conditions to assess if a differential tolerance to starvation stress can explain the differences observed in routine growth conditions. The chosen starvation conditions were (i) HOMEM with decreasing concentrations of FCS and (ii) PBS only. Parallel cultures in routine conditions (HOMEM + 20% FCS at 26°C) were used as controls (Fig. 2, row A).

The cultures in PBS (data not shown) did not grow, just like the culture with 0% FCS. The decreasing concentration of FCS had a marked effect on the growth curve of *L*. *donovani* as seen in the gradually decreasing maximum parasite density reached in stationary phase. However, the 3 strains tested here all had a similar response to FCS starvation showing a similar decrease in maximum parasite density by FCS concentration and an unaffected multiplication rate in the logarithmic phase of their growth curve. The latter was shown by the Akaike's information criterion: it calculated that there is a 95.8% likelihood that a simple model where all hill slopes of the growth curves (representing growth speed) was correct compared to only a 4.2% likelihood that a more complex model with a different hill slope for each curve was correct. These results suggest that the differences seen in the growth curves under normal conditions are presumably caused by differential metabolic needs or a difference in quorum sensing.

#### Oxidative, nitrosative and metal stresses

The survival capacity of promastigotes is not only determined by their nutrient need, but also by their capacity to resist stresses encountered in the sandfly and in the host before they hide as amastigotes inside the macrophages. In this experiment, we compared the response of the 3 selected strains to various stresses *Leishmania* encounters during their development in the sandfly gut and upon transmission to the host. Hydrogen peroxide (H_2_O_2_) is released in the sandfly gut during digestion of heme and is also one of the most important stresses promastigotes are exposed to upon phagocytosis by host macrophages [8]. S-nitroso-N-acetylpenicillamine (SNAP) and 3-morpholinosydnonimine (SIN1) are NO, and NO + O_2_^-.^ donors respectively, and were used to mimic the stress imposed by macrophages while *Leishmania* settles inside them. All 3 strains show a similar susceptibility to H_2_O_2_ at each tested time point. However, their tolerance to H_2_O_2_ significantly increases towards the stationary phase (p = 0.002) which may reflect an improving defence against the oxidative burst released by macrophages immediately after invasion of promastigotes. In contrast, the tolerance to NO and NO + O_2_^−.^ remains stable during stationary phase and is similar for all strains (Fig. 2, row B).

Sb^III^, CuSO_4_ and K_2_Cr_2_O_7_ are all metal compounds known to cause complex oxidative stresses: Sb^III^ paralyses the redox metabolism of *Leishmania*, copper (Cu^2+^) catalyses the reaction of highly oxidative OH^−^ radicals after its reduction to Cu^+^ [28] and the reduction of Cr^VI^ to Cr^III^ causes the formation of an even broader range of oxidative radicals such as O_2_^−.^, H_2_O_2_ and OH^−^ [29]. There were no statistical significant differences in tolerance to Sb^III^ between the strains throughout their growth as promastigotes. The tolerance of BPK282/0cl4 to CuSO_4_ was slightly but significantly higher compared to BPK275/0cl12 at the 4^th^ day in the growth curve, but all strains had a similar susceptibility at day 5 and 6 (Fig. 2, row C). Some notable differences were observed in K_2_Cr_2_O_7_ susceptibility (Fig. 2, row C): BPK282/0cl4 showed a marked decrease in K_2_Cr_2_O_7_ tolerance from log phase towards stationary phase, while the other two strains showed a lower but stable tolerance throughout their growth as promastigotes. This was unexpected, as the strains behaved similarly when exposed to individual oxidants (H_2_O_2_ and O_2_^−.^) that are part of the K_2_Cr_2_O_7_ stress, and thus highlights the complexity of stresses and the responses they elicit in these parasites.

In summary, all strains tested have a similar tolerance to nitrosative and oxidative stress in the promastigote stationary growth phase. Tolerance changes during growth towards stationary phase did occur, indicating that the oxidative/nitrosative stress defence of the parasites might change during the life cycle. The gradually increasing tolerance of all strains to H_2_O_2_ is a form of strict regulation of oxidative stress response during promastigote growth.

### *In vitro* amastigote tests

#### *In vitro* stresses

Reactive oxygen and nitrogen species are extremely important as defence mechanisms of the macrophage against *Leishmania* and also play an important role in the action mechanism of the drug SSG used to treat patients. In fact, SSG imposes a complex pressure on the parasite: (i) its reduced trivalent form Sb^III^ paralyses *Leishmania's* redox metabolism [30], [31] and (ii) SbV enables host cells to re-activate their ROS/RNS attack on the intracellular amastigotes [32]. Here we tested the *in vitro* response of amastigotes to 3 stresses thought to reflect the stresses encountered in the host: (i) SSG, (ii) NO released by SNAP and (iii) NO + SSG by combining SNAP with SSG.

The 3 isolates of which our strains were derived have a well documented variable tolerance to SSG both *in vitro* and *in vivo* [16]: BPK282/0 (SSG-sensitive) and BPK085/0 (SSG-resistant) were isolated from patients who eventually showed definite cure after SSG-treatment, while BPK275/0 (SSG-resistant) was isolated before the onset of treatment of a SSG non-responder patient. The three derived clones used in this study were re-tested and showed a similar *in vitro* SSG-susceptibility as their respective mother-isolates: BPK282/0cl4 was SSG-sensitive and BPK085/0cl3 and BPK275/0cl12 SSG-resistant.

**Figure 3:**
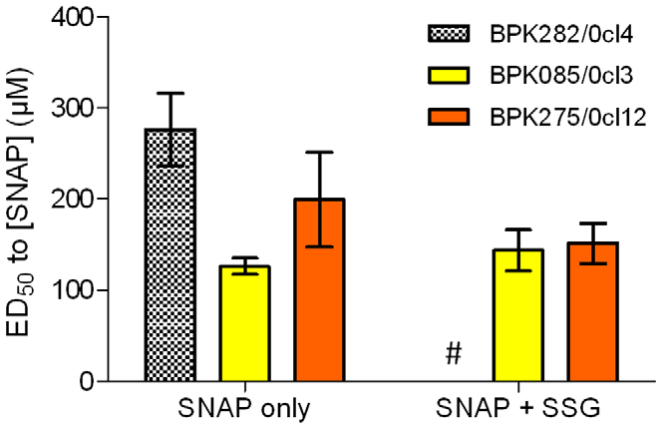
*In vitro* amastigote susceptibility to SNAP and SNAP +60 µg/ml SSG. Error bars indicate the standard error of the mean based on respectively 3 and 2 repeated experiments. # indicates BPK282/0cl4 that was eliminated under the combined pressure of SNAP and SSG. No statistical differences were found. All data is available as supplementary material (Table S2).

The *in vitro* SNAP-susceptibility test in absence of SSG shows variation in NO tolerance, BPK085/0cl3 had the lowest tolerance to NO and BPK282/0cl4 the highest (Fig. 3). In the presence of 60 µg/ml SSG, a dose around 6 times higher than the ED_50_ of the SSG-sensitive strain BPK282/0cl4, both SSG-resistant strains showed a similar susceptibility to NO released by SNAP and BPK282/0cl4, being SSG-sensitive, was eliminated. No statistically significant differences were observed in this experiment although some variation in their response to NO released by SNAP is seen. This variation seemed to diminish in the presence of SSG.

The importance of ROS and RNS in the defence system of the macrophage against *Leishmania* [8] as well as in the action mechanism of SSG led to the hypothesis that SSG-resistance might be linked to tolerance to stresses imposed by the macrophage on the parasite. Several groups have found SSG-resistant *L*. *donovani* to be more tolerant to killing by activated macrophages, hydrogen peroxide (H_2_O_2_) and Sb^III^ when compared to SSG-sensitive *L*. *donovani* [33], [34], or showed that *in vitro* induced Sb^III^-resistant strains were cross-resistant to NO [35]. In this study we could not link the strains' SSG-susceptibility to a higher or lower tolerance to a specific stress *in vitro*. However, *Leishmania* is known as an expert in macrophage and host manipulation, hence it could be that the SSG-resistant parasites found a way to prevent macrophage release of ROS/RNS, which would undermine the SSG activity and at the same time disable the host defence systems. It could be more advantageous for the parasite to prevent a stress from happening than to defend against it. Studies on the interaction between SSG-resistant parasites and the macrophage are therefore also important in the study of SSG-resistance.

Furthermore, *in vitro* models are only a simple representation of the complex environment *Leishmania* encounters in nature. *In vivo* there is a complex interplay between available nutrients, immune system responses and the stresses imposed by the macrophage on *Leishmania.* The absence of an immune system regulating macrophage activity is likely to be a crucial flaw of *in vitro* systems such as the one used here. The latter is also thought to be the reason for the poor predictive value of the *in vitro* determined SSG-susceptibility of Nepalese clinical isolates for the SSG-treatment outcome of the corresponding patients [16]. However, these *in vitro* models are still useful to study amastigotes in simple controllable conditions.

#### *In vitro* infectivity

**Figure 4:**
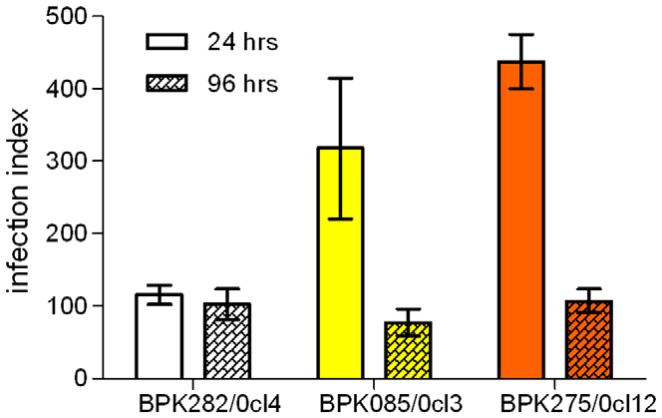
Infection index caused at 24 and 96 hours post infection. Error bars indicate the standard error of the mean based on 3 repeated experiments. BPK085/0cl3 and BPK275/0cl12 cause a significantly higher *in vitro* infection burden at 24 hours post infection compared to BPK282/0cl4. However, no differences between strains were found at 96 hours post infection. All data is available as supplementary material (Table S3).

We infected macrophages *in vitro* with a fixed parasite/macrophage ratio for all strains and evaluated the infection index at 24 and 96 hours post infection to assess their infectivity and infection profile in macrophages. This experiment mimics the initial stages of infection of the macrophage after transmission of *Leishmania* from the sandfly to the host. (Fig. 4). At the early stage of infection (24 hours post infection), BPK085/0cl3 and BPK275/0cl12 show a significant higher infection index compared to BPK282/0cl4 (p<0.05 and p<0.001 respectively). This could be due to a higher percentage of metacyclic parasites in the SSG-resistant strains as compared to the SSG-sensitive strain. Tests on the strains' tolerance to complement lysis, considered as a marker for metacyclogenesis [36], confirm that our SSG-resistant strains indeed have a higher metacyclogenic capacity compared to our SSG-sensitive strain, with stationary phase cultures of BPK282/0cl4 showing a 3-fold lower concentration of complement lysis resistant parasites as compared to BPK275/0cl12 [37]. Metacyclogenesis studies on *in vitro* induced SSG-resistant parasites might provide a further insight into the relation between SSG-resistance and metacyclogenic capacity of *L. donovani*. However, a decrease in both the percentage of infected cells and the amount of amastigotes per infected cell cause the infection index of the SSG-resistant strains at 96 hours post infection to drop to a similar level as BPK282/0cl4, indicating that BPK085/0cl3 and BPK275/0cl12 are apparently not able to fully benefit from their initial advantage in this *in vitro* model. We suspect this is due to the limitations of the *in vitro* model, more specifically the absence of immune system factors which are normally present in an *in vivo* environment. The higher infection capacity during the initial stage of *in vitro* infection seen with SSG-resistant strains might nevertheless have a more persistent character in *in vivo* infection.

#### *In vivo* growth profile

The ultimate approach to compare the capacity to establish infection of the 3 strains is in an *in vivo* environment. Three groups of Balb/c mice were injected with the 3 strains and mice were sacrificed at 7 time points during 2.5 months to evaluate parasite load in bone marrow, spleen and liver samples by relative quantitative PCR.

**Figure 5:**
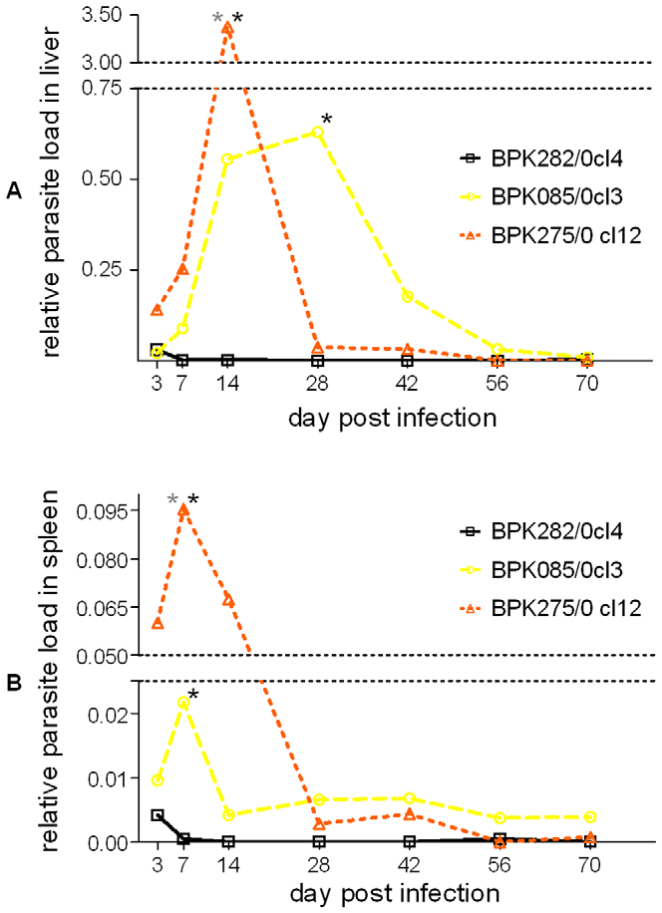
Average relative parasite load per strain over time in liver (A) and spleen (B). Black stars indicate a significant difference with BPK282/0cl4 and grey stars with BPK085/0cl3 at a specific time point. Error bars are not shown to keep the figure as clear as possible, but all data is available as supplementary material (Table S4). BPK275/0cl12 and BPK085/0cl3 cause a marked infection burden in the spleen of Balb/c mice as compared to BPK282/0cl4.

In the liver, BPK282/0cl4 elicits a marked lower infection compared to the two other strains (Fig. 5A); the infection curves of BPK282/0cl4 and BPK275/0cl12 were significantly different (p = 0.0134) by two-way ANOVA. Similarly as in the liver, BPK282/0cl4 seems to elicit a lower infection burden in the spleen as well (Fig. 5B). Here, all infection curves were significantly different from each other (BPK282/0cl4-BPK085/0cl3: p = 0.0456; BPK282/0cl4-BPK275/0cl12: p = 0.0041; BPK085/0cl3 and BPK275/0cl12: p = 0.0178).

The area under each curve is an indicator for the total parasite load in liver and spleen over time, and further supports the observation that BPK085/0cl3 and BPK275/0cl12 have the capacity to cause a more severe infection *in vivo* as compared to BPK282/0cl4 (Table 3).

**Table 3:** Area under curve (AUC) data of liver and spleen infection burden over time.

Strain	Liver AUC	Spleen AUC
BPK282/0cl4	0.113	0.018
BPK085/0cl3	18.130	0.448
BPK275/0cl12	38.050	1.459

The majority of the bone marrow samples remained negative throughout the whole period of sampling. Only one mouse infected with BPK085/0cl3 (at day 28 post infection) and 4 mice infected with BPK275/0cl12 (at day 3 (n = 1), day 7 (n = 1) and day 14 (n = 2) post infection) had a positive bone marrow sample. None of the bone marrow samples taken from BPK282/0cl4 infected mice were positive at any time point. No further analysis was done on the bone marrow samples since the positive sample size was too limited.

In general, BPK282/0cl4 caused the lowest parasite burden in mice, BPK275/0cl12 the highest and BPK085/0cl3 had an intermediate profile. The SSG-resistant strains caused a marked higher infection burden in mice compared to the SSG-sensitive strain. This higher parasite load could not only lead to an increased potential of transmission by the sandfly but also to a more severe disease, making it more difficult for any treatment to eliminate the parasite in the patient. Others have shown that patients that do not respond to SSG-treatment, have a higher parasite load before treatment compared to patients responding well to SSG [38]; a superior capacity of SSG-resistant strains to establish *in vivo* infection could be one of the underlying mechanisms of this observation. Our *in vivo* data also confirms the higher infection capacity seen *in vitro* at 24 hours post infection, but does not support the drop in infection level observed *in vitro* at 96 hours post infection. Since the *in vivo* model is much closer to reality, even taking the limitations of a mice model into account, we suspect that the *in vitro* decrease in infection towards 96 hours post infection was a consequence of the limitations of the *in vitro* model used here. The observation that SSG-resistant parasites survive longer in a model that is known to be self-healing through ROS and RNS pressure on the parasite suggests that these SSG-resistant parasites are able to better prevent or have a higher tolerance to the complex mixture of ROS and RNS they encounter *in vivo*, indicating the relevance of these stresses in the mechanism of SSG-resistance.

### Conclusion

In our model, three Nepalese *L. donovani* strains were evaluated and compared on the level of their *in vitro* and *in vivo* behaviour and their tolerance to *in vitro* stresses. In general, BPK085/0cl3 and BPK275/0cl12 attained stationary phase at a higher parasite density, contained a higher amount of metacyclic parasites and had a greater capacity to cause *in vivo* infection in mice compared to BPK282/0cl4. These traits correlate well with the strains' *in vitro* susceptibility to SSG, and partially with *in vivo* SSG-treatment outcome, leading to the hypothesis that SSG-resistant strains have a better chance for survival and transmission compared to the SSG-sensitive strain. This hypothesis needs to be further experimentally verified and currently a validation study on a larger number of strains is being done for this purpose. If further experiments indeed proof to support this hypothesis, *L. donovani* would be one of the first organisms to our knowledge showing an increased fitness instead of the usual fitness cost [39] after acquiring resistance to a drug. This unusual epi-phenotype is likely the result of the unique combination of an unusual drug (SSG) which relies on the defence mechanisms of the host and an unusual parasite (*L. donovani*) which is highly adaptive. One might say that, after adapting to the host defence system in its early evolution, SSG-resistant *L*. *donovani* overcame ROS and RNS exposure for the second time in its evolution to overcome SSG-treatment of the patients. However, the impact of other drugs on the biological fitness of these parasites still needs to be explored.

Due to the high toxicity and reported failure of SSG-treatment, Miltefosine (MIL) is now the official first line treatment against VL in the Indian subcontinent. This action eliminates, or at least severely decreases, the SSG-drug pressure on the parasite population. However, this will not necessarily abolish the hypothesised increased *in vivo* fitness of SSG-resistant parasites if they are indeed less susceptible to the immune system of the host. Surviving longer in the host gives these parasites more time to adapt to pressure imposed by other treatments, especially drugs with a long half life such as MIL. As for now, MIL still has an acceptable treatment success rate in the Indian subcontinent, but a correlation between the widespread SSG-resistance and a relatively higher tolerance to MIL has already been suggested *in vitro* [40]. Combining our observations with this study suggests that it is probably only a matter of time before MIL-resistance and treatment failure will develop in the Indian subcontinent. Given this possible impact of SSG-resistant parasites with superior fitness on VL epidemiology and control, it is crucial that future studies specifically address the fitness of SSG-resistant parasites and assess the survival and prevalence of SSG-resistant parasites in the parasite population which is now under Miltefosine pressure.

## Supporting Information

Table S1 Data *in vitro* promastigote stress tests.
(0.03 MB XLS)

Table S2 Data *in vitro* amastigote stress tests.
(0.01 MB XLS)

Table S3 Data *in vitro* infection.
(0.02 MB XLS)

Table S4 Data *in vivo* infection.
(0.02 MB XLS)
